# Systemic lupus erythematosus in a patient with multiple system atrophy: A case report

**DOI:** 10.1097/MD.0000000000018005

**Published:** 2019-11-15

**Authors:** Dimitrios Pilalas, Georgios Chatzopoulos, Georgia D. Kaiafa, Eleftheria Ztriva, Soultana Spyridonakou, Vasiliki Bisbinas, Panagiotis Ioannidis, Apostolos I. Hatzitolios, Christos Savopoulos

**Affiliations:** a First Propedeutic Department of Internal Medicine; b Second Department of Neurology, Medical School, Aristotle University, Thessaloniki, Greece.

**Keywords:** case report, fever, multiple system atrophy, systemic lupus erythematosus

## Abstract

**Rationale:**

Multiple system atrophy is a late-onset rare neurodegenerative movement disorder which results in debilitating disease. Fever frequently ensues in the context of infections which can be associated with significant morbidity and mortality, but among alternative diagnostic possibilities neoplasms and autoimmune disorders should be considered.

**Patient concerns:**

We describe a case of a prolonged febrile syndrome in a 55-year-old female patient with onset of multiple system atrophy two years before presentation. Patient history and symptoms were not contributive to guide the diagnostic work-up.

**Diagnosis:**

Initial evaluation provided no specific findings. Repeat testing of auto-antibodies revealed positive antinuclear and anti-ds DNA antibodies coupled with low complement which in conjunction with renal biopsy substantiated the diagnosis of systemic lupus erythematosus flare.

**Intervention:**

Pending the biopsy result, treatment with hydroxychloroquine and corticosteroids was initiated. Due to failure to achieve remission, azathioprine was added, but symptoms persisted. Following the diagnosis of lupus nephritis, azathioprine was discontinued and induction treatment with cyclophosphamide in accordance with the Euro-Lupus regimen was initiated and upon completion followed by maintenance therapy with mycophenolate mofetil.

**Outcomes:**

The patient achieved remission after cyclophosphamide was added to treatment with corticosteroids and has not experienced new flares during the next two years. The neurological syndrome has remained stable during this period.

**Lessons:**

To our knowledge, we report the first case of concurrent systemic lupus erythematosus and multiple system atrophy. Prolonged fever presents unique challenges in patients with rare diseases.

## 1 Introduction

Multiple system atrophy(MSA) is a neurodegenerative movement disorder with orphan disease status as it affects approximately 0.6 patients per 100,000 population every year.^[1]^ The most frequent presenting symptoms include parkinsonism, cerebellar ataxia and autonomic dysfunction in a patient in the 6th decade of life; symptoms progress with debilitating effects and death ensues after a mean duration of 8 years.^[2]^ The disease is sporadic but familial cases have also been described, and the genetic and epidemiological correlates of the disease are not clearly defined.^[1]^

Our current understanding of the pathogenesis of multiple system atrophy is incomplete and the central event in the cascade is the aggregation of α-synuclein and the formation of glial cytoplasmic inclusions which leads to oligodendrocyte dysfunction and release of misfolded α-synuclein extracellularly. The inflammation and the impaired oligodendrocyte function lead to neuronal dysfunction and precipitate cell death. The misfolded α-synuclein exhibits prion-like properties and can spread to other brain areas. Depending on the affected site the clinical presentation varies: striatonigral degeneration presents as parkinsonism with poor response to levodopa, olivopontocerebellar atrophy with cerebellar syndrome and degeneration of the brain stem and the medullary autonomic nuclei with failure of the autonomous system.

In our report, we describe a 55-year-old female MSA patient, presenting with a persistent febrile syndrome, who was diagnosed with systemic lupus erythematosus (SLE).

## 2 Case presentation

A 55-year-old female patient of Greek origin was admitted in our hospital due to a recurring febrile syndrome up to 39^o^C during the week before the admission for which the family physician had prescribed ciprofloxacin for presumptive urinary tract infection in the context of intermittent bladder catheterizations without resolution of the symptoms.

The patient was diagnosed with probable MSA with onset 2 years before presentation. Initially, urinary incontinence was attributed to genitourinary syndrome of menopause and due to failure to manage with medical treatment the patient resorted to intermittent bladder catheterization. Symptoms progressed with frequent falls and documentation of orthostatic hypotension and finally, parkinsonism. The disease was not responsive to levodopa treatment and a DaT scan was not consistent with Parkinson's disease.

Patient history was also notable for asthma, hypothyroidism, depression, placental abruption, and endoscopic resection of multiple benign colon polyps.

At presentation the patient was unwell without any specific complaints and febrile with marked orthostatic hypotension and normal oxygen saturation. Clinical examination was unremarkable except for the neurological status where no modifications with respect to the patient's baseline were noted. The complete blood cell count on admission was notable for normocytic anemia with low reticulocyte count (Hemoglobin 9.8 g/dl). Renal function was normal and hypokalemia (K 2.9 mEq/l) as well as hyponatremia (Na 128 mEq/l) with low specific gravity as a surrogate for urine osmolality and low urine sodium (21 mEq/l) were documented. Serum lactate was normal. A chest X-ray demonstrated limited infiltrations in the left lower pulmonary field.

The patient was admitted and due to the overall clinical status hydration with isotonic fluids and treatment with piperacillin/tazobactam was initiated in the absence of positive inflammatory biomarkers (erythrocyte-sedimentation rate, C-reactive protein and procalcitonin). Repeated blood and urinary cultures were negative and due to the persisting febrile syndrome, without evidence of focus of infection a total body computed tomography (CT) scan and a transthoracic ultrasound were conducted without contributive findings. The infectious diseases work-up came back negative; despite negative antinuclear antibodies and anti-double stranded (ds) DNA antibodies, the complement was low. Serum protein electrophoresis documented presence of a multiclonal gammopathy and a bone marrow biopsy was not contributive.

Due to the persistence of fever in face of a clinical deterioration of the patient after 2 weeks of hospitalization new sets of cultures were drawn and the antimicrobial treatment was escalated. Repeat urinalysis was positive for proteinuria in the absence of an active sediment. In 24 h-urine collection, a total protein of 783 mg was quantified. A repeat evaluation for antinuclear antibodies was positive with a titer of 1:640 and positive anti-ds DNA along with low complement. Given the neurological status, we conducted brain magnetic resonance imaging (MRI) which did not reveal abnormal findings and it was decided not to proceed with lumbar puncture. Taking into consideration the complexity of the case and the presence of proteinuria a renal biopsy was deemed necessary and documented mesangial proliferative glomerulonephritis, thus, substantiating the diagnosis of systemic lupus erythematosus flare. Pending the biopsy result, treatment with hydroxychloroquine and corticosteroids (equivalent of methylprednisolone 1 mg/kg/day) was initiated. Due to failure to achieve remission, azathioprine was added, but symptoms persisted. Following the diagnosis of lupus nephritis, azathioprine was discontinued and induction treatment with cyclophosphamide in accordance with the Euro-Lupus regimen was initiated. Corticosteroids were tapered gradually. The patient responded after the first cycle of cyclophosphamide and was discharged after 40 days of hospitalization, but fever recurred five days before the 2nd cycle. A second hospitalization followed and after the third cycle there was no recurrence of the fever. Upon completion of the Euro-Lupus regimen, maintenance therapy with mycophenolate mofetil was initiated. Treatment was well tolerated and the patient has remained on complete remission two years after diagnosis.

## 3 Discussion

We present a patient with probable MSA who was diagnosed with SLE manifesting as persistent febrile syndrome. Although our patient fell short of fulfilling the classic criteria of fever of unknown origin (FUO) as clues to the diagnosis were provided by the low complement and the autoantibodies, the prolonged febrile syndrome without substantial elements to guide the differential diagnosis except for the lack of chills in patient with multiple potential foci of infection due to her debilitating disease.^[3,4]^

The intermittent bladder catheterization in the setting of neurogenic bladder dysfunction is a well-established risk factor for urinary tract infection but the diagnosis was not corroborated.^[5]^ Neither was a respiratory tract infection, despite the potential for micro-aspirations in a bedridden patient with deterioration of her neurological disease.^[6]^ Other infectious causes were ruled out through reevaluation of the patient history and a targeted work-up. Nevertheless, the prolonged fever of the patient along with episodes of hypotension given the impaired autonomic system response in a context of high prevalence of nosocomial infections and increased antimicrobial resistance, mandated the use of broad spectrum antibiotics and the escalation of the antimicrobial treatment.

The work-up we undertook did not provide evidence to a neoplastic origin of this febrile syndrome, but clues to an auto-immune disorder. Fever as a manifestation of SLE occurs frequently but it rarely is the sole manifestation of SLE at presentation and frequently it responds to corticosteroids.^[7]^ Given the deterioration in the clinical status and the proteinuria in the absence of other manifestations to substantiate the diagnosis coupled with the presence of anti-ds DNA antibodies,^[8]^ we resorted to renal biopsy which corroborated the diagnosis of SLE in accordance with the Systemic Lupus International Collaborating Clinics classification criteria (SLICC).^[9]^

Systemic lupus erythematosus is a multisystem autoimmune disease notorious for its protean manifestations,^[10]^ whereas a definitive diagnosis of multiple system atrophy can only be made through brain biopsy.^[2]^ Thus, we considered the possibility that the constellation of symptoms in our patient may be in part manifestations of systematic lupus erythematosus.^[11]^ We believe that the clinical course of the patient spanning years before the flare, the lack of abnormal findings in the MRI of the patient and the lack of response of the neurological syndrome to a treatment regimen that induced and maintained long term remission reasonably exclude the possibility that the principal components of the neurological syndrome are associated with SLE.

In our literature search, we found no previous reports of co-existence of multiple system atrophy and systemic lupus erythematosus and current evidence do not seem to lend support to a shared pathogenesis.

## Author contributions

**Conceptualization:** Dimitrios Pilalas, Georgia D. Kaiafa, Christos Savopoulos.

**Data curation:** Georgios Chatzopoulos, Eleftheria Ztriva.

**Formal analysis:** Dimitrios Pilalas, Christos Savopoulos.

**Funding acquisition:** Georgia D. Kaiafa.

**Investigation:** Georgios Chatzopoulos, Eleftheria Ztriva.

**Supervision:** Apostolos I. Hatzitolios, Christos Savopoulos.

**Validation:** Georgia D. Kaiafa, Soultana Spyridonakou, Panagiotis Ioannidis.

**Writing – original draft:** Dimitrios Pilalas, Vasiliki Bisbinas.

**Writing – review & editing:** Georgia D. Kaiafa, Soultana Spyridonakou, Panagiotis Ioannidis, Christos Savopoulos.
